# Covid-19 Protesters and the Far Right on Telegram: Co-Conspirators or Accidental Bedfellows?

**DOI:** 10.1177/20563051221129187

**Published:** 2022-10-25

**Authors:** Cliona Curley, Eugenia Siapera, Joe Carthy

**Affiliations:** University College Dublin, Ireland

**Keywords:** far right, COVID-19, Telegram, topic modeling, social network analysis

## Abstract

The COVID-19 pandemic led to the creation of a new protest movement, positioned against government lockdowns, mandatory vaccines, and related measures. Efforts to control misinformation by digital platforms resulted in take downs of key accounts and posts. This led some of these protest groups to migrate to platforms with less stringent content moderation policies, such as Telegram. Telegram has also been one of the destinations of the far right, whose deplatforming from mainstream platforms began a few years ago. Given the co-existence of these two movements on Telegram, the article examines their connections. Empirically, the article focused on Irish Telegram groups and channels, identifying relevant protest movements and collecting their posts. Using computational social science methods, we examine whether far-right terms and discourses are present and how this varies across different clusters of Telegram Covid-19 protest groups. In addition, we examine which actors are posting far-right content and what kind of roles they play in the network of Telegram groups. The findings indicate the presence of far-right discourses among the COVID-19 groups. However, the existence of these groups was not solely driven by the extreme right, and the incidence of far-right discourses was not equal across all COVID-19 protest groups. We interpret these findings under the prism of the mediation opportunity structure: while the far right appears to have taken advantage of the network opportunity structure afforded by deplatforming and the migration to Telegram, it did not succeed in diffusing its ideas widely among the COVID-19 protest groups in the Irish Telegram.

In early 2020, the potential impacts of the newly emerging COVID-19 pandemic began to penetrate the national consciousness in Ireland, as was the case globally. Major sporting, social, and commercial events were canceled, and schools were closed. In March 2020, following the example of other countries in Europe, the Irish government announced the unprecedented decision to initiate a national lockdown.

By the end of March 2020, a small number of Irish groups and channels focused on dissent from official COVID-19 measures emerged on Telegram, a social media platform characterized by a hands-off content moderation policy. Participants in some of the groups became involved in organizing protests in Dublin, and later across Ireland. Over the next 15 months, these Irish COVID-19 protest channels and groups increased both in terms of quantity and levels of participation.

As the pandemic continued, the key focus of these movements moved from protesting lockdowns to concerns around mask wearing and school closures, and eventually to a strong anti-vaccination stance. Irish media outlets reported concerns held by law enforcement (Hosford and Moore, 2021; Lally, 2020) and researchers (Gallagher & O’Connor, 2021a, 2021b) in relation to the presence of far right personalities, and the narratives that they espoused, within these messaging groups. Given that the main social media platforms (Meta Platforms, YouTube, and Twitter) had “deplatformed” far right actors (Rogers, 2020) and sought to remove or suppress COVID-19 misinformation, questions arise as to the ability of COVID skeptics to emerge as a coherent social movement, as well as the relationship of this movement to far-right actors.

A primary task of this study is therefore to examine content posted in Irish COVID-19 Telegram groups during the pandemic and establish its connections to the far right. In particular, using computational social science methods, we examine whether far-right narratives are present and how this varies across different clusters of Telegram groups. This research also looks at which actors are posting far-right content, and in turn, what kind of roles these actors play in the network of Telegram groups. This is undertaken to provide a clearer understanding of the potential level of influence of far-right narratives within the COVID-19 protest groups.

The findings indicate the presence of far-right narratives among the COVID-19 groups. However, the existence of these groups was not solely driven by the far right. In particular, the research uncovered a clear presence of far-right terms most prominently in one cluster of groups. It also found that actors involved in promoting these terms, when compared with the overall data set gathered, had posted on a higher number of COVID-19 protest groups and were more likely to be administrators of at least one COVID-19 protest group. We interpret these findings under the prism of the mediation opportunity structure, showing that while the far right had made use of the network opportunity structure to build and expand its reach, it did not succeed in taking advantage of the discursive opportunity structure to diffuse its terms and frames among the COVID-19 groups.

## Social Media, COVID-19, and the Far Right

The Internet was embraced by the far right at a very early stage, with the use of bulletin boards and websites. For example, Daniels (2009) identified the ways in which Internet discussion for a breathed new life into White supremacist and far-right groups, which profited from the lower costs of publishing and circulating materials. The rise of social media actively contributed to the dissemination and mainstreaming of far-right content and hate discourses, including racism and white supremacy, xenophobia and anti-migrant hate, anti-feminism and anti-LGBTQ content (Fielitz & Thurston, 2018; Ging & Siapera, 2019; Mudde, 2019; Winter, 2019). In addition, research has shown that far-right groups have successfully used the Internet for mobilization and coordination, with occasional spill over street violence (Johnson, 2018; Müller & Schwarz, 2021).

Studies that focused on digital media have outlined the responsibilities of platforms (Flew, 2018; Gillespie, 2018), which have responded with tighter content moderation policies and more careful enforcement. In the European Union, the European Commission entered a voluntary agreement with platforms to control illegal hate speech and misinformation through the Code of Conduct and Code of Practice, respectively. These agreements include regular monitoring of the efficiency of the policies and their enforcement (Jourová, 2019; Kirk & Teeling, 2019). Such regulatory developments, coupled with events such as the “Unite the Right” rally in Charlottesville in 2017 and the storming of the US Capitol in January 2020, have contributed to a deplatforming of far-right personalities and groups from mainstream digital platforms such as Facebook, Twitter, and YouTube. This in turn is associated with a shift toward alternative platforms, which have less stringent content moderation policies (Rogers, 2020).

Examining alternative platforms, often referred to as Alt Tech, Donovan et al. (2018) assert that such platforms have become central to the recruitment and organizing efforts of the far right. They argue that the US-based far right responded to the mass deplatforming following the Charlottesville rally by mobilizing a tactical innovation (McAdam, 1983), which in this context involved migrating to Gab, a new social media platform with the explicit purpose and design to provide an infrastructure to the alt and far right (Donovan et al., 2018). Alt Tech, therefore, mobilizes specific affordances that cater to the needs of social movements that find themselves excluded from the mainstream, including privacy settings, the use of anonymity, and minimal content moderation alongside standard features of social media such as instant messaging, voice, image and video sharing, and live streaming.

During the COVID-19 pandemic, official measures such as lockdowns, restrictions, and mass vaccination programs were to an extent met with skepticism, and in some cases, outright dissent. Street protests organized using social media drew hundreds and sometimes thousands to the streets in protests that occasionally turned violent, prompting action by social media platforms. For instance, a street protest against lockdowns and vaccination in Dublin on 27 February 2021 resulted in injury to three members of An Garda Siochana (police) and 23 arrests. The Garda Commissioner Drew Harris stated that An Garda Siochana was working with Facebook to identify the individuals involved and to take down pages that violate Facebook’s terms of service (Mahon, 2021). In March 2021, the Irish Tanaiste (deputy prime minister) Leo Varadkar wrote to Facebook, YouTube, Twitter, and TikTok asking the platforms “what more you can do to prevent the use of your platform to promote illegal gatherings and the spread of misinformation?” and “what more can you do to stop the spread of false information on vaccines?” (cited in McDermott, 2021). This has led to a removal of many such accounts from mainstream platforms, which is likely to be a factor in the increasing presence of such groups on alternative platforms and in particular on Telegram. Supporting this argument, de Keulenaar et al. (2020) showed that YouTube adapted its content moderation policies to the spread of COVID-19 misinformation and was increasingly and pro-actively removing this content. These changes were associated with a trend of deplatformed content appearing on numerous alternative platforms, including Telegram (de Keulenaar et al., 2020).

In contrast to the mainstream platforms, alternative platforms seem to have allowed such contents to proliferate freely. For example, news reports indicate that anti-mask groups organized campaigns against mask wearing in Irish schools using Telegram (McNamee, 2021). In the United Kingdom, there were reports of increasingly violent language used against scientists and health workers among anti-vaccination groups on Telegram (Yeomans et al., 2022). In Germany, authorities considered banning the app, because of its role in organizing and staging protests in politicians’ houses and calling for people to “share private addresses of German ‘local MPs, politicians and other personalities’ who they believed were ‘seeking to destroy’ them through pandemic curbs” (Agence France Presse, 2021).

Since COVID-19 protest groups and the far right are both regularly deplatformed by the mainstream platforms and hosted by alternative tech, questions emerge concerning their connections, and their ideological and tactical alliances. While COVID-19 groups appear to borrow ideas, rhetoric, and tactics from the far right (Gallagher & O’Connor, 2021a), we lack more detailed information on the extent and direction of their relationship with the far right. To what extent is COVID-19 skepticism driven by the far right?

While research of far-right activity on Telegram has offered considerable insights on far-right movements, there is little research on the activities of COVID-related groups on Telegram and their links to the far right. On the other hand, research on the links between anti-lockdown, vaccine skepticism, anti-masking rhetoric, and the far right has tended to focus on mainstream social media, such as Twitter. For example, research by Caiani et al. (2021) on British and Italian far-right politicians and their COVID rhetoric on Twitter found that COVID offered them the opportunity to reframe their traditional friend and enemy categories while also reinforcing their relationship with their bases.

In 2020, Telegram became increasingly popular with Irish far-right groups and their followers. Research by Gallagher and O’Connor from the ISD found 60,377 messages posted by 34 Irish far right channels in 2020. This compared to their findings from 2019 in which they discovered just over 800 messages in a few Irish channels. The ISD report also noted that groups and individuals associated with the far right in Ireland were central to the organization and promotion of anti-mask and anti-lockdown protests in 2020, and that almost 1 in 10 messages posted by Irish anti-lockdown and COVID-19 conspiracy theory on Telegram channels originated from far-right sources (Gallagher & O’Connor, 2021a).

While studies such as Gallagher and O’Connor (2021a) provide useful insights into the links between the far-right and COVID-19 protest groups, important questions remain unanswered. To what extent do far right groups drive COVID-19 protest narratives? How do COVID-19 protest groups relate to far-right rhetoric and ideas? And how can we make sense of these links theoretically? Are these two distinct socio-political movements or have they coalesced into one? These questions are at the center of this article. Before formulating our research questions, we discuss the mediation opportunity structure, a key concept from social movement theory which constitutes the theoretical framework for our analysis.

## Social Movements and Mediation Opportunity Structure

A key question in political science and in particular in social movement studies concerns the role played by external socio-political structures in facilitating or hindering political mobilization. A central focus is the identification of which structures enable or prevent political groups from successfully mobilizing for social change. The term political opportunity structure is used to capture any “signals to social or political actors which either encourage or discourage them to use their internal resources to form social movements” (Tarrow, 1996: 54). For Tilly (1978), one of the main theorists of this approach, political opportunity reflects an alignment between the interests of a group, the world around it, and the options, risks, and threats, available for collective action.

**Figure 1.**
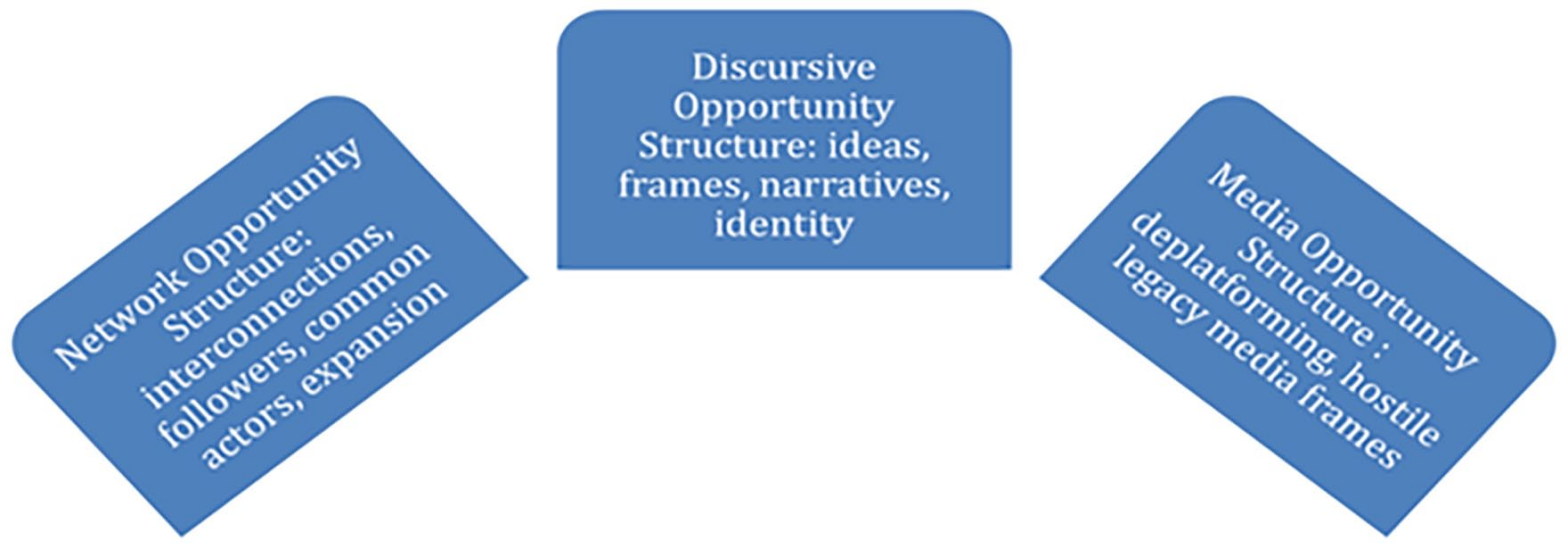
Mediation opportunity structure for far right and COVID-19 movements, after Cammaerts (2012).

Expanding the notion of political opportunity structure, Cammaerts (2012) developed the concept of a mediation opportunity structure. This seeks to address the question of the role of the media in helping or hindering the development and success of social movements. The political and mediation opportunity structures are semi-independent from one another: while the political opportunity structure refers to the socio-political conditions under which social movements are formed, the mediation opportunity structure explores the communicative structures that facilitate the emergence and growth of a social movement and support its construction of a movement identity. Cammaerts posits that the mediation opportunity structure has three components: (1) a discursive opportunity structure, which refers to the production of ideas, narratives, and frames associated with a movement; (2) a media opportunity structure, which refers to the representation or framing of the movement by legacy media; (3) a network opportunity structure, which refers to the affordances and ways in which social media platforms enable a movement to grow or conversely hinder its development (Figure 1).

Both the political and mediation opportunity structures are used by researchers to identify challenges and action possibilities for social movements. It is crucial to note that such structures constitute potentialities that protest movements can take advantage of, or in other words, they constitute the context within which movement agency can operate. Using the mediation opportunity structure and more specifically its two components of discursive opportunity and network opportunity, we seek to understand how the far-right and COVID-19 movements use Telegram, an alternative social media platform. In particular, we consider deplatforming and the generally hostile legacy media position toward both movements as a media opportunity structure that challenges both movements. Following the mediation opportunity structure schema, transfer to another platform, Telegram, is expected to present discursive opportunities for (or challenges to) developing ideas, narratives and frames, and ultimately a stronger identity for the movement. In addition, Telegram is expected to create network opportunities for (or challenges to) growth, sharing of resources, actors, and followers. Does Telegram present a mediation opportunity structure that leads to the confluence of the two movements? What opportunities for growth and the development of common frames and identity do movement actors follow? What influence, if any, do far-right actors have within the COVID-19 movement? Using the mediation opportunity structure, we therefore formulate the following research questions:

RQ1: What are the main themes and frames emerging from COVID-19 protest groups on Telegram? To what extent do these converge or diverge from far-right narratives?
RQ2: What role do the main actors posting far-right content in the COVID-19 protest groups play within the overall COVID-19 protest movement?

## Research Design and Methodology

To address the research questions, the study combined computational methods with an interpretative approach. In particular, to address RQ1, we extract the most important feature terms, visualize these with word clouds, and use topic modeling to identify the main themes present. To address RQ2, we search for an agreed lexicon of terms and employ social network analysis. In this section, we explain in more detail our approach.

### Data Collection

Data for this study were collected from Telegram, a cloud-based messaging platform accessed through its mobile app which has now been downloaded over one billion times. Telegram has mainstream use globally but has been co-opted by White nationalists and other extreme right users in recent years, and especially after their deplatforming from mainstream social media (Owen, 2019; Rogers, 2020; Urman & Katz, 2022).

Telegram comprises numerous entities, including users, chats, supergroups, and channels. Channels are used to broadcast messages to large audiences and can have an unlimited number of subscribers. Chats are used for group discussions. Supergroups are also discussion groups, but these can have up to 200,000 members. Supergroups can be public or accessible by invitation only (Telegram, 2021a). Supergroups and groups will be referred to in this article collectively as groups.

In April 2020, during the early days of the COVID-19 pandemic, one of the authors of this article discovered an Irish Telegram channel entitled “End the Lockdown Ireland” which was dedicated to organizing protests against recently implemented lockdown measures. This channel described itself as a noticeboard, and a link to local groups protesting the lockdowns and associated government measures. “End The Lockdown Ireland” was selected by the authors as the initial seed channel to be applied in an Exponential Discriminative Snowball Sampling method, as is appropriate for selecting a data set in a hidden population involved in sensitive activities (Etikan et al., 2016). All posts were collected for this channel with code written in Python, utilizing Telethon and other Python libraries, and querying the Telegram Application Programming Interface (API). The data collection and research approach received ethics clearance by the authors’ university.

Links to other Telegram groups and channels were extracted from the posts of the seed channel. These were assessed independently by two members of the research team, and an agreed list of those that were deemed to be Irish-based and related to COVID-19 protest were selected for the first round of data collection. All posts were collected for selected groups and channels. Links to additional groups and channels were extracted from these posts in turn, assessed in the same manner, and the posts of further Irish COVID-19 protest groups and channels were retrieved in a final round of snowball sampling. Supergroups that were accessible by invite were joined only if linked from other channels or groups, and accessible to all by clicking on a link.

Data were collected over a 2-week period in May 2021. This time period included the pre-processing that was required to map URLs to groups or channels so that each could be assessed for each subsequent round of data collection. Data were stored in a combination of PostgreSQL database tables and comma-delimited files.

In total, 330,688 posts and related metadata of 112 Telegram entities were collected. These posts were retrieved from 6 groups, 19 channels, and 87 supergroups. The data included engagement figures and role of posters (administrator or participant). For ethical reasons, no data are reported that could be attributed to individual users. To address RQ1, we performed a content analysis using a topic modeling method. To address RQ2, we searched for an agreed lexicon of terms and performed social network analysis. We explain below how the methods were applied.

### Content Analysis Through Topic Modeling

Each of the 112 channels and groups was labeled as belonging to one of eight groupings, as outlined later in Table 2 in the “Results” section. This clustering was undertaken to get a better understanding of the topics that were being discussed, given the diverse nature of the groups and channels. Some clear clusters emerged based on their own self-identification and labeling, for example, the national and regional channels and groups relating to “The Resistance” or “IRE” protest movements. Others, for example, channels or groups dedicated to being points of information were grouped together following manual review of the title, “about” statement (where available) and first five text posts of each one. The size and other characteristics of each grouping, or cluster, are detailed in the “Results” section of this article.

The data set was filtered so that analysis would only include text posts. Posts that had no text or that were merely “Action” posts, a record of the action of setting up or joining a channel, were excluded. This resulted in a total of 264,661 posts available for analysis across eight cluster groupings. A Term Frequency-Inverse Document Frequency (TF-IDF) model, which rates how important each word is relative to all words in the corpus, was then applied to the text of posts from each cluster, to extract the most important feature terms. The parameters of this model were set to ignore terms that appeared in more than 1% of the posts to ensure that the most common terms were excluded. The rarest terms, which appeared in less than five posts, were also excluded. The research team built a tool to experiment with different minimum and maximum parameters for excluding terms, each time outputting which terms would be lost if either parameter is changed. It was clear from these experiments that over-exclusion of the rarest or most common terms would in effect “throw out the baby with the bathwater.” Terms such as “patriots” or “sheeple” that might be relevant to far right narratives would be excluded alongside non-specific terms that could be associated with numerous different topics.

As an alternative strategy, and in addition to optimizing the TF-IDF maximum and minimum terms parameters, four stopword lists were built to bolster the standard stopword list from the Python NLTK library and to exclude common terms. This strategy ran the risk of preventing the model from producing bigrams relevant to far right narratives, such as “indigenous irish” or “Kalergi plan” if, for example, the common terms “irish” or “plan” were ignored. To minimize any losses of relevant terms, the lists of stopwords were reviewed by two members of the research team. Supplemental Appendix 1 contains a description of the stopwords used.

Code was written using the sklearn, MATLAB, and wordcloud Python libraries to calculate the TF-IDF of the extracted terms from posts retrieved from the database and to plot the most frequent bigram terms as a wordcloud for each cluster of groups and channels. Pre-processing was also conducted on the data before vectorization to remove special characters, single characters, multiple spaces and convert to lowercase.

The Latent Dirichlet Allocation (LDA) algorithm was then employed to create topic models which enabled the research team to identify the most important topics in the posts for each cluster of Telegram groups and supergroups. LDA can be used to train a model which extracts topics as collections of terms from a corpus, and this model can be applied to assign one or more of these topics to every document (in this case every post) in the corpus. Each topic consists of multiple terms, which are weighted in relation to their importance for the topic. These terms can be shared among different topics.

The LDA method developed by Blei et al. (2003) was used in this study because it can handle mixed-length documents. However, it does require a predefined number of topics, and if this is not chosen correctly, topics may be too generalized or may overlap (Albalawi et al., 2020).

Prior to applying the LDA algorithm on each cluster of posts, TF-IDF was undertaken to achieve the richest dataset for topic modeling with terms that expose thematic content (Blei & Lafferty, 2009). We extracted unigrams (single-word terms) that were present in more than three documents but less than 10% of the documents, to exclude very rare or very common terms.

Two coherence measures, log-likelihood and perplexity, were calculated for running the LDA topic modeling algorithm with *n* = 3, 5, and 10 topics. This was done to assess for each cluster data set, what *n* would retrieve the most coherent set of topics. The aim, therefore, was to achieve the lowest perplexity score possible and the highest log likelihood possible. It was determined that the optimal number was 4. The process is described in more detail in Supplemental Appendix 2.

Once the optimal number of topics was agreed to be *n* = 4, a topic model was generated as previously discussed with this parameter. The results, including top 20 words for each topic, and the number of documents (posts) in which each topic was the most dominant topic, are outlined in Supplemental Appendix 3. These have been run for each cluster of groups and channels and were manually labeled by the research team.

Having examined the content of the groups and channels through TF-IDF, wordclouds, and topic modeling, the research team decided to exclude broadcast channels from the next phase of analysis for the following reasons. First, broadcast channels, although technically a similar entity to groups (Telegram, 2021b), offer only one voice, that of the owner of the channel. Others can comment on posts, but in general it is one user. Groups and supergroups are discussion forums, with many users able to contribute. Therefore, they are very different entities in relation to establishing centrality of influencers. It is also not generally possible to uniquely identify the user who owns a channel, only those who comment in a channel. In addition, the TF-IDF analysis, wordclouds and topic modeling of the clusters, indicated a more notable presence of far right narratives and hate speech content in the one cluster (the “Resistance” one), which comprised supergroups only, so this supported the option of narrowing down to groups (including supergroups).

### Identifying Far-Right Actors and Social Network Analysis

To address RQ2, the research must identify far-right actors in the overall network. To this end, we looked at potential methods to identify posts containing far-right messaging. There are numerous challenges in this regard. First, how does one define far-right messaging? What criteria can be used to identify this? Searching for specific terms, even where these can be agreed upon, can obviously produce false positives. For example, even unambiguous references to far-right narratives such as the “Kalergi Plan,” which refers to a supposed conspiracy to replace white populations with people of color, could be used in a critical context, and not necessarily in support of this theory.

Unsupervised methods such as topic modeling can be very useful to provide a broad overview of topics within a large data set. A topic model once trained can be applied to a data set, with each document (post) assigned to one or more topics. However, initial efforts by the research team in applying a topic model to label a dominant topic for each post in the data set were in many cases inconclusive or had assigned a topic inappropriately, particularly for the shortest posts which may only have a few words of welcome to the group, to give one example. Future research may focus on the methodological challenges of identifying far-right content as secondary or hidden topics in online discussion groups.

In the present study, we sought to identify potential far-right content through a data classification method of searching for terms from the Hatebase lexicon. This was narrowed down to searching for unambiguous terms to decrease the likelihood of mis-identification of far-right content. However, the Hatebase lexicon is limited to racist and hate speech terms, which are useful indicators of far-right content, but do not necessarily cover other far-right discourses such as White replacement.

For this reason, the research team supplemented the Hatebase search terms with other far-right terms that were identified by applying a Bag of Words (BoW) model extended with TF-IDF, which rates how important each word or phrase is relative to all words in the corpus. This extracted bigrams and trigrams appeared in between .001% and 1% of the posts. There are potentially hundreds of thousands of bigram and trigram phrases in such a big corpus, hence the use of the min and max parameters to cut out most common and most rare phrases. A stopword list, as outlined previously but extended with COVID-related terms, was also used to refine the list of terms sufficiently for manual review.

Two members of the team independently reviewed the bigrams and trigrams list and agreed on a shortlist of terms that were indicative of far-right narratives. These narratives are outlined in Table 1 and exclude COVID-19 conspiracies.

**Table 1.** Far-Right Narrative Categories and Associated UnambiguousTerms.

Narrative	Description	Examples
Replacement	terms associated with the far right “theory” that white people are being replaced.	“population replacement genocide”
Racism	racist and hate-based terms.	“blm antifa”
Legal	references to laws revered by the far right, nazi trials or unconstitutionality of current systems	“nuremberg code”
Anti-elite	terms referring to anti-elite conspiracies	“agenda 2030”

Using the combined Hatebase and BoW approach, the number of posts to be reviewed was further reduced to 255,999 text posts from groups and supergroups in the data set. A search was conducted on the reduced data set of the posts of 93 groups and supergroups for 362 unambiguous terms from the Hatebase lexicon and 16 unambiguous terms from the reviewed TF-IDF BoW. Posts that contained one or more of these terms were extracted from the data set.

The relationship of groups and clusters to the posts containing the extended list of terms was then visualized on Gephi using the ForceAtlas2 algorithm. ForceAtlas2 is a “force-based” algorithm, that is, nodes generally repulse each other, and edges attract their nodes (Jacomy et al., 2014).

For each actor identified as posting content containing terms in the Hatebase lexicon (extended with the 16 terms), the following was then examined:

1. Privileges (Admins vs Participants): Whether they have elevated roles in the groups in which they have posted the hate-based or extreme content, that is, administrators, creators, or first posters in at least one of the groups.
2. Presence (Superusers vs Users): The number of groups or supergroups in which actors have posted, and whether this number is less than or equal to the number of channels in which the 95th percentile of actors has posted.

The relationship of actors to the extended terms was then similarly visualized on Gephi using the ForceAtlas2 algorithm. The following section presents our findings.

## Results

As outlined in the previous sections, a key concern of this study is to understand the relationship between the COVID-19 movement and the far right. Using the mediation opportunity structure, we focus on discourse and networks to identify the extent to which the discourses overlap, meet, or diverge, and the extent to which far-right actors have any influence in the COVID-19 groups under study.

### Overview of Collected Data

**Figure 2.**
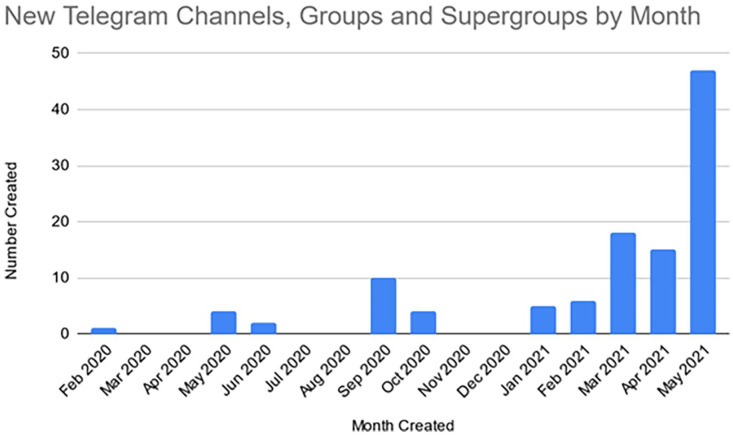
Timeline of the creation of Irish COVID-19 protest entities on Telegram.

Figure 2 shows the number of new Irish COVID-19 protest channels and groups that were created on Telegram each month between February 2020 and May 2021. This reveals a sharp increase in entities in May 2021, 34 of which can be attributed to one particular cluster of supergroups.

Of the 34 groups set up during May 2021 (noting that data collection took place in the period 7–21 May 2021), 30 of these were set up by one individual user. Creator and Admin user details are not available for some groups (or any channels). Users that could be identified from the data as having created at least one channel had in the main created only that one channel. However, 50% of the channels in which a creator could be identified were created by just two users.

In terms of general participation levels, this ranged between 4 and 4,346 participants per group or channel, with median 73 and an arithmetic mean of 238 participants per group or channel. However, it should be noted that some groups or channels were in existence for a much longer period (up to 15 months) when the data were collected. Others were created merely days or weeks before the data were collected.

**Figure 3.**
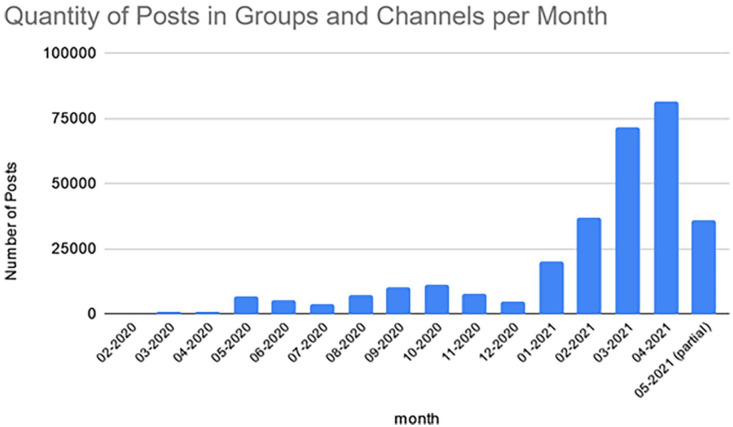
Number of posts posted each month.

In total, 330,688 text posts were collected in the 112 Telegram entities, including comments on posts. If one excludes “action” posts, such as setting up a channel or a new member joining, and posts that contain no text, for example, where image or video media was attached or forwarded but without any commentary or web link, this figure reduces to 264,661. Figure 3 maps out the posting of this content over a 15-month period. Although data were collected for only part of May 2021, it is clear from this bar chart that there was a significant increase in postings in the early months of 2021.

Table 2 shows details and characteristics of the eight clusters into which the data set of channels and groups was divided for comparison purposes. These were not of equal size in terms of numbers of posts or participants. Some were clustered together based on their own self-identification, for example, the national and regional channels and groups relating to “The Resistance” or “IRE” protest movements. Others were grouped together following manual review of the title, “about” statement (where available), and first five text posts of each one. Some clusters contain a mixture of channels, groups, and supergroups, while others contain only one or two of these entity types.

**Table 2.** Group and Channel Clusters.

Cluster name	Cluster description	Members	Number of text posts	Number of participants
ATCG	National and regional “ATCG” or Anti-Tyranny Citizen Groups and channels.	2 channels 11 supergroups	7,564	457
Children	Groups and channels that are concerned with parenting or the education of children	1 channel 2 groups 8 supergroups	29,795	2,178
Freedom	Groups and channels that highlight personal freedom, including medical freedom and freedom of speech	4 channels 2 groups 3 supergroups	10,490	4,663
Info	Channels and groups focused on information sharing	8 channels 2 groups 9 supergroups	29,329	5,703
IRE	National and regional groups for the “IRE” (Ireland) nationalist movement	34 supergroups	57,157	3,929
Resistance	National and regional groups for “The Resistance”	5 supergroups	26,753	562
Voicechat	The only group dedicated to voice chat	1 supergroup	54,015	4,346
Other	Channels and groups that do not fit easily into the above clusters	4 channels 16 supergroups	49,558	4,810

Our analysis focuses on comparing activity within the eight clusters outlined above, with the aim of identifying whether far-right narratives could be seen across the entire data set or were restricted to specific clusters.

## Contents: A Common Discourse?

Do COVID-19 groups contain far-right posts? How widespread are far-right posts among COVID-19 Telegram groups? This section presents the findings of the content analysis and topic modeling. To set the context for this analysis, the section begins with a descriptive approach using wordclouds containing bigrams for each of the clusters under study. This is followed by the topic modeling per cluster.

**Figure 4.**
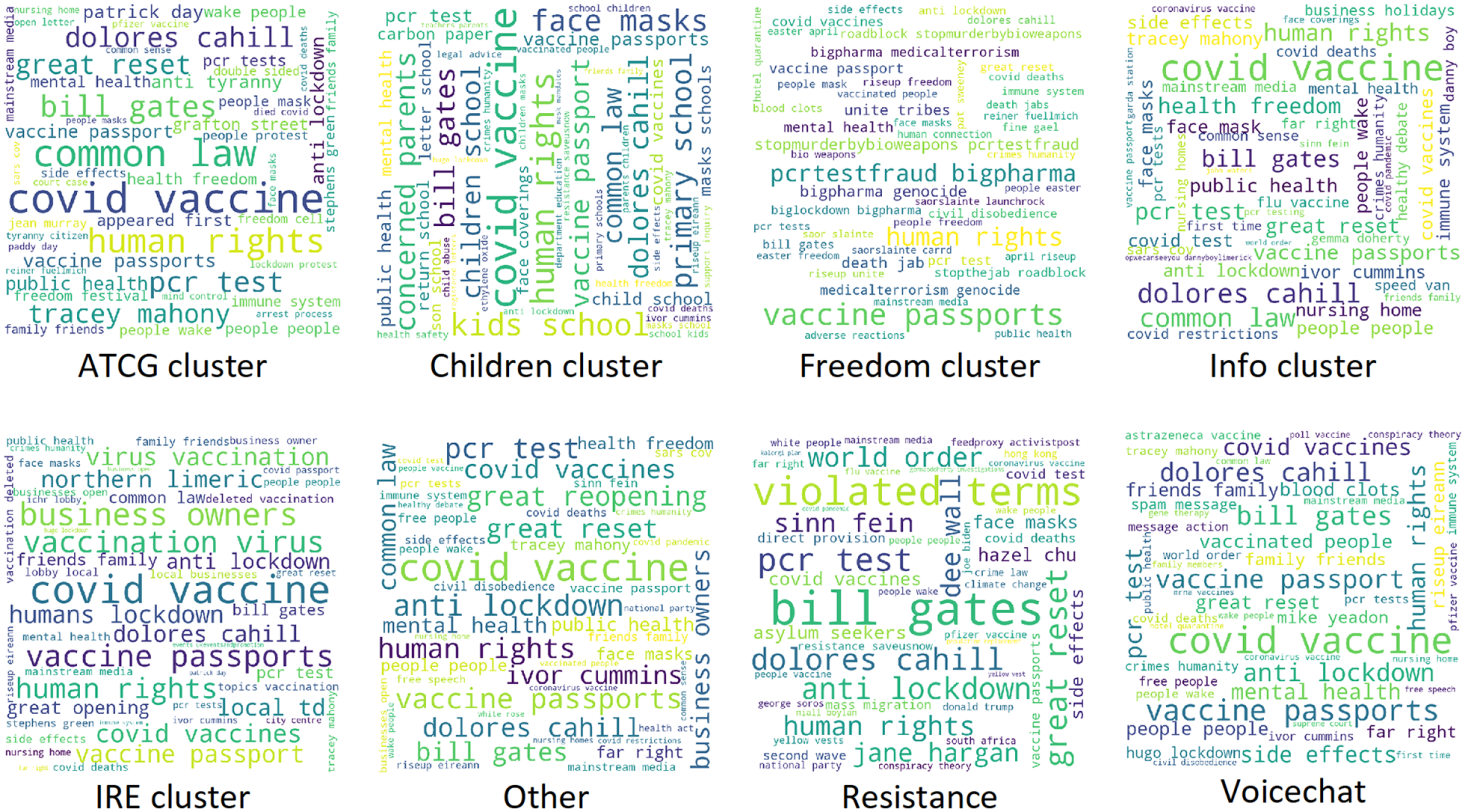
Wordcloud representation of most frequent 50 bigrams for each cluster of groups.

Wordclouds can provide a useful indication of topics discussed in a collection of online posts, by displaying the most frequent terms with a font size relative to their own frequencies. By grouping the Telegram entities into broadly similar clusters, a picture emerges of some key discussions within the group. For instance, the wordcloud representation for most of the clusters show common COVID-19-related terms such as “covid vaccine” or “vaccine passport,” as might be expected. Common to most groups are also terms that could be associated with COVID-19 conspiracy theories such as the “great reset” or “bill gates.” A marked difference occurs in only one grouping, the “Resistance” cluster which displays terms that are commonly associated with far-right narratives such as “asylum seekers,” “kalergi plan,” “white people,” and “mass migration.” Although it is recognized that some of these terms could be used in a non-extreme context, their presence in a COVID-19 protest group is unexpected (Figure 4).

In addition, some key personalities, and in particular Dolores Cahill, are present in almost all the clusters. Cahill, a former professor at the School of Medicine in University College Dublin, became a key figure in the COVID-19 skeptic movement in Ireland and globally, after disputing the severity of COVID-19, and taking a stance against vaccination and mask wearing. Until March 2021, Cahill was the chair of the far-right Eurosceptic Irish Freedom Party. While the bigrams provide an indication of the presence of far-right ideas and terms in COVID-19 protest groups, a more detailed analysis through topic modeling can delve deeper into any connections.

### Topic Modeling by Cluster

Topic modeling using the LDA algorithm was used to extract four topics for each cluster as outlined in the methodology section. The results, including top 20 words for each topic, and the number of documents (posts) in which each topic was the most dominant topic (where one topic was dominant), are outlined in Supplemental Appendix 3. Topics related to COVID-19 emerged strongly as expected. However, anti-establishment and populist topics emerge most prominently in the ATCG, Freedom, and Resistance clusters. The Resistance cluster, detailed in Table 3, appears to have a particular focus on anti-establishment and “resistance” themes, and the appearance of topic words that reflect far right narratives appears to be higher. These topic words include “antifa,” “blm,” “war,” “biden,” “bill,” “gates,” “resistance,” and “agenda.” However, it should be acknowledged that these words could appear in non-extreme contexts.

**Table 3.** Topics and Topic Words Found in the Resistance Cluster for *n* = 4.

Cluster	Topic labels	Number of posts (dominant topic)	Dominant topic frequency	Top 20 words
Resistance	Anti-establishment	4,869	0.181998	terms, violated, gpo, point, god, view, john, working, minutes, change, antifa, days, court, bill, name, gates, friend, corona, war, town
	Protest	5,066	0.189362	protests, mobile, friends, message, deaths, looks, die, far, action, jane, dee, biden, times, important, sense, hargan, family, tonight, normal, death
	Anti-establishment	5,012	0.187343	check, agenda, shit, pandemic, week, nice, fake, garda, job, face, blm, june, bad, water, safe, fantastic, bitcoin, poll, million, kids
	Resistance	5,044	0.18854	brilliant, meet, school, city, mass, kids, fuck, open, excellent, jean, numbers, resistance, pfizer, conspiracy, letter, local, lockdowns, course, schools, fight

This analysis shows first that not all COVID-19 clusters contain far-right posts, and second, that the far-right posts appear most frequently in one cluster, the “Resistance” one.

### Terms Search

Since topic modeling cannot provide very detailed information on the spread of far-right posts across all groups under study, we performed a term search, to assess the frequency of far-right terms. An initial search of the posts for only the terms found in the Hatebase lexicon returned 156 unique posts in 28 groups posted by 61 actors. After the list of search terms was extended to include the additional terms identified by the research team, this new search returned 1,568 posts across 63 groups posted by 460 unique actors. Supplemental Appendix 4 contains a table with some examples of these posts. The results of these searches are detailed in Table 4.

**Table 4.** Results of Using Hatebase and Additional Terms for Searching Posts From Groups and Supergroups Across All Clusters.

	Number of instances	Number of unique posts	Number of unique posters	Number of unique groups	Number of unique terms
Hatebase terms only	163	156	61	28	16
Extended terms only	1,405	1,268	432	62	16
Both sets of terms	1,568	1,382	460	63	32

To understand better the distribution of these posts across the clusters, we performed a social network analysis on the contents.

**Figure 5.**
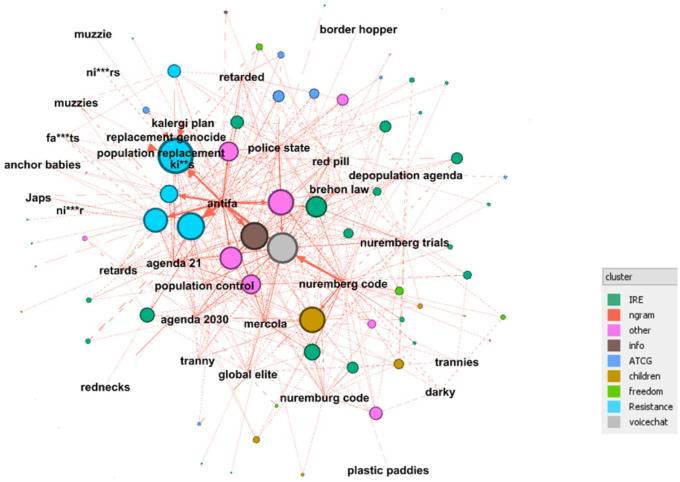
Bipartite graph of group clusters and relationship with unambiguous Hatebase terms extended with other far-right bigrams.

The graph in Figure 5 visualizes mentions of terms from the Hatebase lexicon (extended with BoW terms) across 93 Irish COVID protest groups. The nodes in this bipartite graph are the groups and the terms associated with hate speech or other far-right narratives. The 93 groups and supergroups are grouped into clusters as previously outlined (with channels removed), and these are displayed in different colors on the graph, according to cluster. Terms which would generally be considered to be highly offensive are redacted, except where this would hinder recognition of a term.

The ForceAtlas2 algorithm has been applied to the node and edge files to highlight linked nodes. Node size is dependent on the indegree of each node. Therefore, larger nodes are the ones that have had the most mentions of one or more of these terms. The teal-colored nodes which belong to the Resistance cluster are prominent given that its five supergroups represent only approximately 10% of the posts and approximately 5% of the participants in the overall data set (excluding channels).

In response to the first research question, concerning the extent to which a common discourse between the far-right and COVID-19 protest groups emerges, our findings from the content analysis, including the topic modeling, the term search, and social network analysis of the terms, indicate a modest presence of far-right terms and by extrapolation ideas and discourses among the COVID-19 groups. In particular, it was mainly concentrated in one of the clusters we identified, the self-proclaimed “Resistance” cluster. This modest presence suggests that while COVID-19 and deplatforming of skeptic groups from mainstream platforms created a discursive opportunity structure for the far right, this did not ultimately lead to the production of a common narrative linking the two movements.

## Far-Right Actors and Their Potential Influence

**Figure 6.**
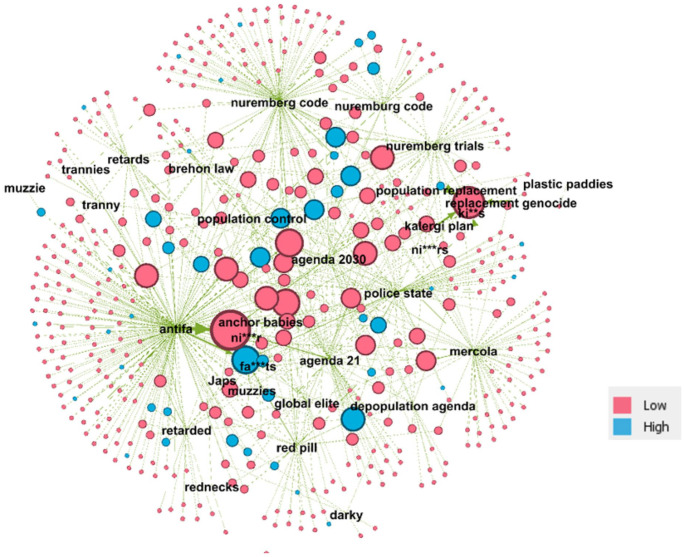
Bipartite graph of actors and relationship with unambiguous Hatebase terms extended with other far right bigrams highlighting level of privilege.

In this section, we address the second research question, concerning the role of far-right actors in the COVID-19 protest groups. As outlined previously, identification of actors posting far-right or hate content was based solely on text search on the text posts of groups or supergroups. This was because it was not possible to compare the activity of actors in channels and groups in a “like for like” manner, since groups contain posts from multiple participants, whereas only channel admins will post in channels. Focusing therefore on groups, in this section, we examine the extent to which those posting far-right contents were integral or peripheral actors in the COVID-19 groups. To examine this, for each actor identified as posting content relating to hate terms and/or far-right narratives, Figure 6 highlights levels of privilege. We define privilege as having at least one elevated role in the groups in which they have posted the hate-based or far-right content such as administrator, creator, or first poster.

Node size is relative to the number of posts for that actor which were returned from the extended terms search. Nodes are colored pink if actors are participants only in the relevant groups. If actors have one or more elevated roles, then nodes are colored blue.

Of the 460 actors we identified as posting far-right contents (see Table 4), 61 have one or more elevated roles in the group(s) in which they have posted the aforementioned content, a rate of approximately 13%. When compared with all participants in the entire data set of groups (excluding channels), the probability of a participant having one or more elevated roles in a group is less than 2%. What is also notable is the node size of many of the blue nodes, suggesting that a significant proportion of those that were responsible for multiple identified posts also had one or more elevated roles, and hence a potentially greater level of influence. This shows that the far right posts were not random posts from random users but came from users who had roles of potential importance in this milieu.

To compare the activities of actors who post far-right content to the entire data set of participants of COVID-19 groups, we examined the number of groups in which far-right actors have posted, and whether this number is less than or equal to the number of groups in which the 95th percentile of overall group participants has posted. In total, 11,550 unique actors participated in the 93 groups. The number of COVID-19 protest groups in which each of these participants have posted was calculated and the arithmetic mean was found to be 1.87 groups, with a standard deviation of 2.02 and interquartile range (IQR) of 1. Ninety-five percent of participants were found to have posted in five groups or less.

The average number of groups in which each of the 460 far-right actors had participated was also calculated. In this small subset of the overall data set of participants, far-right actors had each participated in an average of 4.94 groups, with standard deviation of 4.48 and an IQR of 5, suggesting that these actors had a significantly higher presence in the Telegram COVID-19 protest group ecosystem.

To gauge the influence of the far-right actors, in addition to looking at the likelihood of these actors having one or more elevated roles, we also examined the average number of groups in which they have posted. We then compared this to the average number of groups in which all participants in the data set had posted.

**Figure 7.**
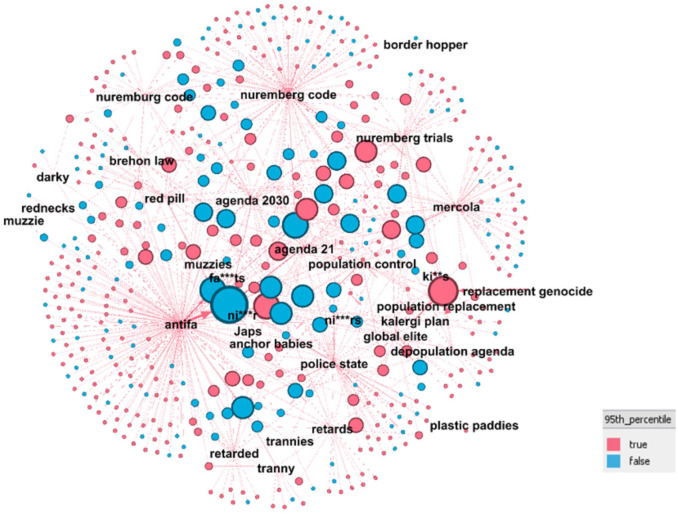
Bipartite graph of actors and relationship with unambiguous Hatebase terms extended with other far-right bigrams highlighting number of groups in which the actors have posted.

Figure 7 uses colors to represent which of the 460 identified actors were posting in a number of groups less than or equal to the 95th percentile of the data set as a whole. Blue nodes represent those who have participated in more than five groups, that is, those outside the 95th percentile of the overall data set of participants. Thirty-one percent of actors fall into this category, as opposed to 5% in the overall data set.

In this social network graph, node size is again relative to the number of relevant posts made by an individual participant. Therefore, the largest nodes represent those who have published the most extreme or hateful posts, as defined earlier. This social network graph also demonstrates a potentially higher level of influence for the far-right actors due to their higher presence in multiple channels relative to average levels of presence across the entire sample group.

These findings suggest that the network opportunity structure, understood as the opportunity to use social networking media to grow and recruit more members, is taken up by far-right actors, who are actively posting and engaging with COVID-19 protest groups to a greater extent than is normal for participants in these groups.

## Conclusion

This article is concerned with identifying the extent to which the COVID-19 protest groups coalesced with the far right on Telegram. We discussed literature that showed that both COVID-19 protest groups and the far right were deplatformed from mainstream media, driving them toward platforms such as Telegram. Literature that has studied the far right has identified narratives that seek to connect to COVID-19 protests (Caiani et al., 2021) but the extent to which COVID-19 groups contain or engage with far-right contents has not been examined, and this is what the present study sought to do. Theoretically, we used the framework of the mediation opportunity structure (Cammaerts, 2012), which posits that movements mobilize by making use of discursive opportunity structures to develop and disseminate counternarratives; network opportunity structures, which help them grow and recruit members; and media opportunity structures, by which mainstream media pick up on the movement discourses and demands. Focusing on the first two within the context of the Irish Telegram groups and channels, we asked: to what extent has the far-right and COVID-19 protest groups developed a common narrative? How influential are far-right actors among the COVID-19 groups?

Our findings indicate that while far-right contents are present in the COVID-19 groups, they are not very widely diffused or embedded across all clusters within this milieu. This suggests that the two movements have not developed a common and cohesive narrative and that far-right contents are marginal within the COVID-19 protest movement in Ireland. Using the mediation opportunity structure terminology, the discursive opportunity structure created by their coexistence on Telegram was not sufficient to generate a common overarching counternarrative connecting the two movements. However, looking at the actors who post far-right contents, we found that they tend to have a higher degree of privilege, defined as positions of influence in the groups, such as administrator or creator, and a higher degree of presence, defined as posting across a higher number of groups. This suggests that actors associated with far-right contents are more active and that they are pursuing network opportunities seeking to grow their movement and recruit others.

These findings add nuance to the theoretical perspective of mediation opportunity: while Cammaerts (2012) identified movements where both the discursive and the network opportunities were picked up by movements, in our case, we found an uneven relationship between the two. This in turn suggests that while Telegram exposes users to far-right contents, this does not necessarily translate to recruitment or persuasion. However, we should not take this to imply that we should be complacent. Our study collected data within a relatively limited period of time from the beginning of the pandemic in March 2020 until 21 May 2021, with the first few months of 2021 being the most active. It is possible that continuous and systematic posting of far-right contents among these groups may eventually make them acquire a stronghold. Indeed, Cammaerts’ schema suggests that activities within the network opportunity structure feed into the discursive opportunity structure and vice versa. It is expected in other words that network opportunities will lead to a greater diffusion of discourses and ideas connected to the movement.

In the case of Irish COVID-19 protest groups on Telegram, this did not happen during the period under study, suggesting that there is a window for action: media literacy initiatives or more targeted content moderation policies among platforms such as Telegram may be used to neutralize efforts by the far right to use the mediation opportunity structures to grow and consolidate among other disenfranchised groups on non-mainstream platforms.

## Supplemental Material

sj-docx-1-sms-10.1177_20563051221129187 – Supplemental material for Covid-19 Protesters and the Far Right on Telegram: Co-Conspirators or Accidental Bedfellows? Click here for additional data file. Supplemental material, sj-docx-1-sms-10.1177_20563051221129187 for Covid-19 Protesters and the Far Right on Telegram: Co-Conspirators or Accidental Bedfellows? by Cliona Curley, Eugenia Siapera and Joe Carthy in Social Media + Society

sj-docx-2-sms-10.1177_20563051221129187 – Supplemental material for Covid-19 Protesters and the Far Right on Telegram: Co-Conspirators or Accidental Bedfellows? Click here for additional data file. Supplemental material, sj-docx-2-sms-10.1177_20563051221129187 for Covid-19 Protesters and the Far Right on Telegram: Co-Conspirators or Accidental Bedfellows? by Cliona Curley, Eugenia Siapera and Joe Carthy in Social Media + Society

sj-docx-3-sms-10.1177_20563051221129187 – Supplemental material for Covid-19 Protesters and the Far Right on Telegram: Co-Conspirators or Accidental Bedfellows? Click here for additional data file. Supplemental material, sj-docx-3-sms-10.1177_20563051221129187 for Covid-19 Protesters and the Far Right on Telegram: Co-Conspirators or Accidental Bedfellows? by Cliona Curley, Eugenia Siapera and Joe Carthy in Social Media + Society

sj-docx-4-sms-10.1177_20563051221129187 – Supplemental material for Covid-19 Protesters and the Far Right on Telegram: Co-Conspirators or Accidental Bedfellows? Click here for additional data file. Supplemental material, sj-docx-4-sms-10.1177_20563051221129187 for Covid-19 Protesters and the Far Right on Telegram: Co-Conspirators or Accidental Bedfellows? by Cliona Curley, Eugenia Siapera and Joe Carthy in Social Media + Society
