# Supplementary material for: Covid-19 Protesters and the Far Right on Telegram: Co-Conspirators or Accidental Bedfellows?
Source: Soc Media Soc. 2022 Oct 25;8(4):20563051221129187. doi: 10.1177/20563051221129187 (PMC9597280; doi:10.1177/20563051221129187)
Supplement: sj-docx-1-sms-10.1177_20563051221129187 – Supplemental material for Covid-19 Protesters and the Far Right on Telegram: Co-Conspirators or Accidental Bedfellows? [file sj-docx-1-sms-10.1177_20563051221129187.docx]

**Appendix 1 – Stopword List**

| List name | Description | # Words | Sample words (n=10) |
| --- | --- | --- | --- |
| common | Words that are appearing in the text that are common and do not have any decipherable relevance to far-right narratives | 940 | 'able', 'about', 'absolutely', 'accept', 'accepted', 'access', 'according', 'across', ‘acting’, ‘active’ |
| uncommon | Words that are appearing in the text that are uncommon and do not have any decipherable relevance to far-right narratives | 64 | 'abend', 'dqqycjqrbvzcj', 'ein', 'eine', 'einem', 'einen', 'es', 'ffs', 'guten', 'healthfreedomireland' |
| place | Words relating to place names | 96 | 'america', 'american', 'antrim', 'armagh', 'asia', 'athlone', 'australia', 'belfast', 'belgium', 'bray' |
| urls | Technology, platform and url related stopwords | 155 | 'admin', 'admins', 'admit', 'amp', 'app', 'apps', 'archive', 'article', 'bbc', 'bitchute' |
