# Supplementary material for: Covid-19 Protesters and the Far Right on Telegram: Co-Conspirators or Accidental Bedfellows?
Source: Soc Media Soc. 2022 Oct 25;8(4):20563051221129187. doi: 10.1177/20563051221129187 (PMC9597280; doi:10.1177/20563051221129187)
Supplement: sj-docx-2-sms-10.1177_20563051221129187 – Supplemental material for Covid-19 Protesters and the Far Right on Telegram: Co-Conspirators or Accidental Bedfellows? [file sj-docx-2-sms-10.1177_20563051221129187.docx]

**Appendix 2 – List of Topics**

For all cluster groupings, n= 3 topics resulted in the optimal perplexity and log likelihood scores, followed by n=5. A qualitative assessment comprising visual inspection of the topics was undertaken. This visual inspection examined which n (number of topics) produced the most coherent topics that were relatively straightforward to label, while minimising overlap between topics identified. It was assessed that for all clusters, n=10 produced the least coherent topics with most overlap.

In order to further optimise the number of topics used in the modelling, this process was repeated with n = 3,4 and 5 topics.
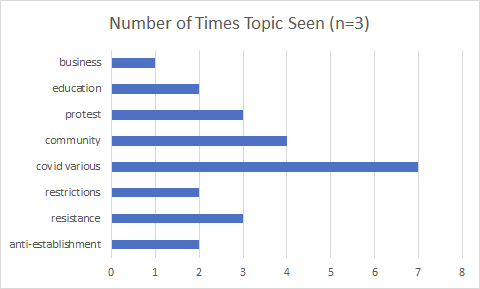


Topics identified for n =3 topics

Although the lowest perplexity and log likelihood scores were achieved for n=3 topics, it was deemed on visual inspection that the least overlap of topics occurred at n=4. Topics were also easier to label, with less requirement for the more generic label of “COVID various” as can be seen in Figures 6 above and Figures 7 and 8 below.


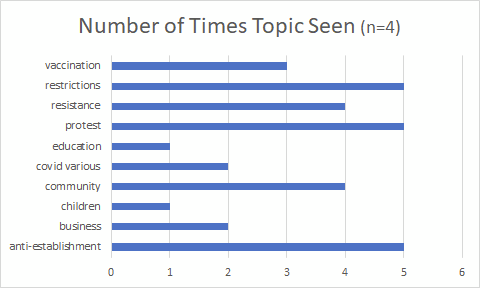


Topics identified for n=4 topics


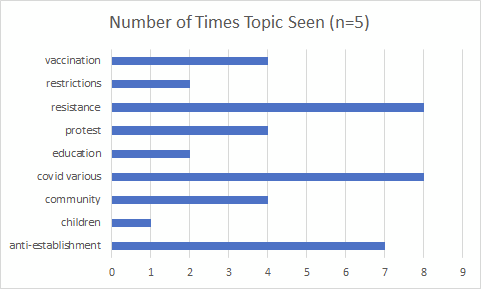


Topics identified for n=5 topics
