# Supplementary material for: Covid-19 Protesters and the Far Right on Telegram: Co-Conspirators or Accidental Bedfellows?
Source: Soc Media Soc. 2022 Oct 25;8(4):20563051221129187. doi: 10.1177/20563051221129187 (PMC9597280; doi:10.1177/20563051221129187)
Supplement: sj-docx-3-sms-10.1177_20563051221129187 – Supplemental material for Covid-19 Protesters and the Far Right on Telegram: Co-Conspirators or Accidental Bedfellows? [file sj-docx-3-sms-10.1177_20563051221129187.docx]

**Appendix 3 - Table of Topic Modelling results with n=4 topics**

| **Cluster** | **Topic Labels** | **# Posts (Dominant Topic)** | **Dominant Topic Frequency** | **Top 20 Words** |
| --- | --- | --- | --- | --- |
| info | children | 4464 | 0.171851 | mobile,kids,sfns,cp,parents,letter,nice,alan,garda,name,problem,friend,fuck,crazy,cool,shocking,holidays,important,woman,madness |
|  | restrictions | 4138 | 0.159301 | check,excellent,god,checkpoints,meeting,checkpoint,kris,tonight,morning,road,city,castlebar,shit,speed,waiting,mad,christmas,jean,mind,night |
|  | community | 6038 | 0.232445 | liam,story,fantastic,message,working,needs,course,court,positive,common,fair,times,number,restrictions,members,businesses,protests,sick,vax,anti |
|  | vaccination | 5604 | 0.215738 | brilliant,fact,vaccination,testing,man,pcr,wrong,doctors,sad,hse,doctor,report,safe,flu,job,vaccinated,pfizer,ill,global,looks |
| freedom | anti-establishment | 2260 | 0.215443 | brilliant,god,fair,caroline,check,nicola,beautiful,blood,bringbacknormal,propaganda,banned,astrazeneca,important,side,shocking,breaking,fraud,power,excellent,pandemic |
|  | community | 1604 | 0.152908 | tribes,meet,united,friends,galwayriseup,months,numbers,tds,rise,emails,proof,wait,standup,fuck,city,course,fighting,conspiracy,action,company |
|  | covid various | 1926 | 0.183603 | story,normal,pcrtestfraud,nice,saorslainte,stopthejab,medicalterrorism,looks,roadblock,sign,shedding,problem,chng,music,restrictions,life,poll,positive,book,fauci |
|  | restrictions | 2048 | 0.195234 | fantastic,fact,control,march,morning,fight,friend,lockdowns,sick,change,members,wake,trust,folks,pauline,anti,needs,fast,travel,plan |
| other | covid various | 10613 | 0.200582 | story,cases,bad,mobile,problem,god,flu,sense,death,march,testing,name,police,jab,tracey,articles,died,hse,fear,woman |
|  | protest | 9362 | 0.176939 | topic,plan,excellent,check,friends,morning,party,working,money,protests,banned,fantastic,needs,town,based,crazy,stickers,michael,ty,important |
|  | business | 12704 | 0.240101 | fair,case,contact,members,message,local,opening,vote,wrong,passports,letter,fatemperor,face,change,vaccination,pcr,school,sad,sign,vaccinated |
|  | restrictions | 8940 | 0.168963 | brilliant,nice,fake,truth,fuck,shit,job,voice,garda,looks,deleted,reopening,sheep,numbers,mind,anti,tonight,course,night,wait |
| ATCG | community | 1325 | 0.175172 | friends,kids,wake,weekend,tracey,night,leaflets,trust,friend,excellent,town,important,working,printed,suits,school,system,normal,reset,job |
|  | covid various | 1323 | 0.174907 | brilliant,looks,business,mercy,needs,cool,kind,safe,learn,vivien,street,head,times,vax,sign,name,travel,patrick,speech,abuse |
|  | protest | 1255 | 0.165918 | jean,dolores,course,scariff,march,level,positive,protests,citizen,members,david,woman,fight,deleted,spread,cahill,spam,city,ref,rules |
|  | anti-establishment | 1625 | 0.214833 | nice,awake,wrong,mind,mobile,case,pcr,action,months,check,gates,removed,court,sick,green,tonight,bill,antifa,plan,tests |
| children | covid various | 5863 | 0.196778 | story,teenheard,problem,check,course,working,deaths,declaration,vax,open,important,date,morning,passport,gates,volunteering,solicitor,messenger,cats,women |
|  | education | 5676 | 0.190502 | mac,campaignemails,primary,shocking,inquiry,needs,looks,members,doctor,sad,dr,friend,document,tests,bad,positive,emails,court,antoin,man |
|  | protest | 5884 | 0.197483 | god,concerned,wake,tusla,campaignletter,campaigninstructions,principal,reply,protest,message,plan,fear,wrong,excellent,template,phone,daughter,hsa,name,teacher |
|  | resistance | 5346 | 0.179426 | brilliant,change,emailed,amhain,testing,registered,paper,write,sign,sentinel,carbon,choice,fair,advice,insurance,nice,police,local,pressure,td |
| IRE | protest | 12180 | 0.213097 | god,town,fear,sad,police,riseup,cool,positive,plan,fuck,garda,sense,poster,test,fight,dolores,square,needs,update,cahill |
|  | community | 11749 | 0.205557 | brilliant,vote,problem,meeting,deaths,green,related,tds,weekend,pfizer,peaceful,excellent,party,morning,safe,change,letters,gates,died,vaccinated |
|  | resistance | 9792 | 0.171318 | deleted,td,topic,nice,topics,left,sign,looks,ichr,lobby,wake,point,side,posters,case,john,street,emails,member,coffee |
|  | restrictions | 11312 | 0.197911 | story,northern,fair,humans,check,city,limeric,friend,rally,school,astandinthepark,night,course,print,based,passports,shit,crazy,mass,fredobwriting |
| voicechat | vaccination | 11470 | 0.212348 | story,jab,bad,tonight,pfizer,friends,test,fake,death,passport,poll,dr,deaths,green,sad,positive,sick,virus,members,needs |
|  | anti-establishment | 8767 | 0.162307 | brilliant,night,fuck,excellent,crazy,shit,change,bill,fight,agenda,sense,deleted,numbers,anti,gates,life,morning,scary,vax,stage |
|  | restrictions | 11155 | 0.206517 | far,dolores,wrong,police,court,support,business,law,state,gardai,man,friend,check,amen,cahill,martin,conspiracy,coronavirus,garda,contact |
|  | resistance | 10194 | 0.188725 | god,point,nice,message,kids,protest,fair,looks,action,wake,iano,money,home,folks,war,spam,shocking,plan,name,head |
| Resistance | anti-establishment | 4869 | 0.181998 | terms,violated,gpo,point,god,view,john,working,minutes,change,antifa,days,court,bill,name,gates,friend,corona,war,town |
|  | protest | 5066 | 0.189362 | protests,mobile,friends,message,deaths,looks,die,far,action,jane,dee,biden,times,important,sense,hargan,family,tonight,normal,death |
|  | anti-establishment | 5012 | 0.187343 | check,agenda,shit,pandemic,week,nice,fake,garda,job,face,blm,june,bad,water,safe,fantastic,bitcoin,poll,million,kids |
|  | resistance | 5044 | 0.18854 | brilliant,meet,school,city,mass,kids,fuck,open,excellent,jean,numbers,resistance,pfizer,conspiracy,letter,local,lockdowns,course,schools,fight |
