# Supplementary material for: Covid-19 Protesters and the Far Right on Telegram: Co-Conspirators or Accidental Bedfellows?
Source: Soc Media Soc. 2022 Oct 25;8(4):20563051221129187. doi: 10.1177/20563051221129187 (PMC9597280; doi:10.1177/20563051221129187)
Supplement: sj-docx-4-sms-10.1177_20563051221129187 – Supplemental material for Covid-19 Protesters and the Far Right on Telegram: Co-Conspirators or Accidental Bedfellows? [file sj-docx-4-sms-10.1177_20563051221129187.docx]

**Appendix 4 – Examples of far right posts identified through the combined Hatebase and BoW methods**

| Let's concentrate on decent people against the lockdown. And to the autists that disagree with this. Go do something useful. Bring an extra 10 people here each. The idea is to grow this group among normal people against the lockdown. Not have it as a sperg echo chamber with less than 100 people. Do you retarded autists want the commies controlling the lockdown movement or do you want a sperg chamber? |
| --- |
| Lots of parents in this group. Most will ignore the fact it's a man. Don't want to deal with it.  Tranny world is where most paedos hide. And duality is a major Tenet of satanism, baphomet the hermaphrodite. Which is why so many trannies in official life relative to their statistical representation in the wider population. They're everywhere, canavan looks like another.  I'm not being religious. But looking at canavan I have to point these things out. |
| Who wants their propaganda movies with virtue signaling, climate change and trannies etc |
| Trannies everywhere because they're so unsure of themselves, easy to control, and serve the double function of worshipping dual sex baphomet or the devil. |
| Just back from walk in Phoenix park. 100s of cars queuing at St.Mary's Hospital to get the genocide jab. Facepalming at all the masked retards sitting in their cars...alone. Dear Aliens, please invade now and nuke this place. We're done here. |
| Remember that the term ‘racism’ was coined & popularised by Lev Davidovich Bronstein (aka Leon Trotsky), a central figure in the Bolshevik movement that butchered the Tsar & his family and led to decades of terror and mass murder in Russia and beyond.  Loyalties to your nation, ethnic group or race must be broken down in order to implement international Communism, as was Bronstein’s main goal. Nations and identities get in the way of that, so a buzzword is created and those not on board with the international revolution can be shamed.  Stop talking about ‘reverse racism’ or ‘the real racists’. Don’t proclaim to be ‘against racism’ (looking at you, CivNat retards). You’re giving legitimacy to the language of Communists.  The word has evolved now. All Whites are ‘racist’. Foreigners all around the world know the power of the word and use it to exploit us.  Don’t argue against the word.  Don’t call your enemies this word.  Don’t be silenced by this word.  Step over it.  Disregard it.  Take away its power. |
| The population replacement genocide of the Indigenous Irish people continues at pace.  Why do we have so many traitors in the Department of Justice waging #KalergiPlan genocide against us in breach of Article II of the 1948 UN Convention on Genocide ?  Why is there no onus on Irish Government Departments to abide by a similar inter-generational contract as the Japanese?  All those foreigners identify as #BAME (Black, Asian, Minority, Ethnic) for the purposes of their own tribal self advancement reintroducing the penal laws into Ireland.  Bone marrow and stem cells are not transferable between the different human races proving that the different human races evolved separately.  ———  People of the 3B_Y (Black, brown, beige and yellow) are born with those skin colours as they’re meant to live in the hot climates of Africa, Asia and South America.  They will always be miserable in Ireland, with its 10 month long Winters.  The only reason that ‘People of the 3B_Y’ are in Ireland is because of the bogus #HolocaustReligion that was created using torture at the Nuremberg Trials.  Irish people were psychologically bullied in to believing in the HoloHoax to accept Multiculturalism, and open borders mass migration by corrupt Kikes like #AlanShatter, #MichaelSmurfit, #StevenSpielberg #GeorgeSoros #TomiReichenthal, and corrupt CryptoKike politicians and Media, and corrupt Brits and West Brits who’d sold their souls to the Kikes, and added their posh respectability to the Holocaust Lie.  The motion for debate is:  ‘#Loxism is the worst form of Wacism in Ireland today.’  Or  The motion for debate is:  ‘The Kikes are a Luciferian people who are at war with nature and the Natural Law, and who are committed to the destruction of the Irish Race, Irish Identity and what it means to be Irish.’  #Scamdemic  https://jrnl.ie/5398353  https://youtu.be/hh_s1pjFvMU  https://t.me/kalergiplan7  https://t.me/InversionAgenda |
| Today (4th of March) is #Justice4TheIrishDay when we call for a safe secure Independent Homeland for the Irish people free from the population replacement Kalergi Plan Genocide.  #Loxism is Wacism  . https://www.dropbox.com/sh/av4t4zyuayezq3s/AABXxn7cZeBy54Whu8LYX7K6a?dl=0  ——————  Why the Jews have spent the past 1,000 years plotting the destruction of the Irish Race.  #ToirdelbachOBrien  The motion for debate is:  ‘It’s time for the K**s and the World Jewish Congress to get all the Africans, Asians, Arabs and South Americans out of Ireland, and back to their own continents.’  #Justice4TheIrishDay  #Ireland4TheIrishDay  https://theirishrising.blogspot.com/2012/08/history-of-jews-in-ireland.html?m=1 |
| Previous minister for Irish children was a gay childless American woman who has since returned to Seattle to join Antifa... This is the new minster for children.. also a childless homosexual who defends pedophilia and cares more about pushing his LGBTP+ agenda across Europe than looking after homeless Irish children  https://twitter.com/rodericogorman/status/1300398156044267521?s=20 |
| Antifa Ireland founder and convicted Pedophile 🤮 with his merry band of child rapists came out to shut down free speech in Dublin this week. 🤡 It's time they drop the "Anti" from their name. |
| Ireland's journalists are waking up to the real issue in our society. Just like the U.S, Antifa and BLM terrorizing cities for months. The real problem in Ireland is radical left wing politics and their hooligan supporters who harassed professional journalists this week in Dublin, Ireland. |
